# Preparation and Evaluation of Aceclofenac Topical Microemulsion

**Published:** 2010

**Authors:** Rohit Ramesh Shah, Chandrakant Shripal Magdum, Shitalkumar Shivagonda Patil, Nilofar Shanawaj Niakwade

**Affiliations:** *Appasaheb Birnale College of Pharmacy, Sangli, India.*

**Keywords:** Aceclofenac, Topical microemulsion, Skin permeation, Phase diagram, Percutaneous delivery

## Abstract

A topical preparation containing aceclofenac was developed using an o/w microemulsion system. Isopropyl myristate was chosen as the oil phase as it showed a good solubilising capacity. Pseudo-ternary phase diagrams were used to obtain the concentration ranges of the oil, surfactant (Labrasol) and co-surfactant (plurol oleique) for microemulsion formation. Five different formulations were formulated with various amount of the oil (5-25%), water (10-50%), and the mixture of surfactant and co-surfactant at the ratio of 4 (45-65%). In vitro permeability of aceclofenac from the microemulsions was evaluated using Keshary Chien diffusion cells with 0.45-μm cellulose acetate membrane. The amount of the aceclofenac permeated was analyzed by HPLC and the droplet size and zeta potential of the microemulsions was determined using a Zetasizer Nano-ZS. The mean diameters of the microemulsion droplets approximately ranged between 154 - 434 nm, and the permeability of aceclofenac incorporated into the microemulsion systems was 3 folds higher than that of the marketed formulation. These results indicate that the microemulsion system studied is a promising tool for percutaneous delivery of aceclofenac.

## Introduction

Aceclofenac [[[2-[(2, 6-Dichlorophenyl)-amino]-acetyl]-oxy]-acetic acid is a non-steroidal anti-inflammatory drug (NSAID) (1). It exhibits a multifactor mechanism of action which is mediated by selective inhibition of prostaglandin E2. The most widely cited side-effect of NSAIDs includes, gastrointestinal ulcer, accompanied by anaemia due to the bleeding, which is also true for aceclofenac. In order to avoid the gastric irritation, minimize the systemic toxicity and achieve a better therapeutic effect, one promising method is to administer the drug via skin (2). Transdermal drug delivery systems provide the most important way to achive these goals (3). The transdermal delivery system also enable controlled or sustained release of the active ingredients and an enhanced patient compliance (4). In this research, formulating topical microemulsions and in vitro permeation studies for aceclofenac was considered.

The concept of microemulsions was first introduced by Hoar and Schulman during 1940s. It is defined as a system of water, oil and amphiphile which is an optically isotropic and thermodynamically stable liquid micro-dispersion (5-7). Microemulsions offer several advantages such as enhanced drug solubility, good thermodynamic stability, ease of manufacturing and enhancing effect on transdermal delivery compared to conventional formulations (7, 8). Water insoluble drugs may be delivered through oil-in-water (o/w) microemulsions (9-11), while water soluble drug may be delivered through water-in-oil (w/o) microemulsions. These systems may also be used for sustained release of drugs by formulating intramuscular preparations (12). 

**Table 1:** Formulations of aceclofenac microemulsion

**Ingredients (in % w/w) **	**AILP-A**	**AILP-B**	**AILP-C**	**AILP-D**	**AILP-E**
Isopropyl myristate	25	20	15	10	5
Labrasol/ Plurol oleique	65	60	55	50	45
Water	10	20	30	40	50

Recently, more attention has been focused on microemulsions for transdermal delivery of drugs. The transdermal delivery of diclofenac (13), diclofenac diethylamine (14), triptolide (15, 16), piroxicam (17), and meloxicam (18) using microemulsions has been reported. 

In this study, we tried to develop a new formulation of aceclofenac in microemulsion base for topical application which may lead an improvement in patient compliance. Microemulsions containing 1% w/w aceclofenac were formulated and examined the diffusion and in vitro skin penetration of aceclofenac from them. 

## Experimental

Aceclofenac BP was gifted by Aarthi Drugs Ltd, Pune. Isopropyl myristate (IPM) was received as a gift sample from Rita Corporation, USA. Labrasol and plurol oleique were gifted by Gattefosse, France. All other chemicals used were of AR grade and used without further purification. 

*Screening of oils, surfactants and co-surfactants for microemulsion formation*

For selecting solvents with good solubilising capacity for aceclofenac, the solubility of aceclofenac was investigated in oils like IPM, IPP, etc and surfactants and co-surfactants like tween 80, plurol oleique, cremophor RH40, labrasol etc. 

An excess amount of aceclofenac was added to 5 mL samples of oils, surfactants, and co-surfactants in screw capped tubes and shaken on orbital flask shaker at 100 RPM for 48 h at ambient temperature. The suspension was centrifuged at a relative centrifugal force of 2795×g and the clear supernatant liquid was decanted and filtered through a 0.45-μm nylon membrane filter (Whatmann). Then, the solubility of aceclofenac was measured by HPLC method. 

*Construction of pseudo-ternary phase diagrams*

The pseudo-ternary phase diagrams were constructed using titration method to determine the microemulsion region and to detect the possibility of making microemulsions with different possible compositions of oil, surfactant/ co-surfactant, and water. 

The ratios of surfactant to co-surfactants were chosen to be 1:2, 1:1, 2:1, and 4:1, and such mixtures were prepared. These mixtures (S/CoS) were mixed with the oil phase to give the weight ratios of 90:10, 80:20, 70:30, 60:40, 50:50, 40:60, 30:70, 20:80 and 10:90. Water was added drop by drop and stirred using a magnetic stirrer until a homogeneous dispersion or solution was obtained. After each addition, the system was examined for the appearance and flow properties. The end point of the titration was the point where the solution becomes cloudy or turbid. The quantity of the aqueous phase required to make the mixture turbid was noted. 

The percentages of the different incorporated pseudo phases were then calculated and the same procedure was followed for the other S/CoS ratios. 

*Preparation of aceclofenac microemulsion*

Aceclofenac was added to the mixtures of oil, surfactant, and co-surfactant with varying ratios as described in Table 1, and then an appropriate amount of water was added to the mixture drop by drop with constant stirring on magnetic stirrer. Microemulsions containing aceclofenac were obtained spontaneously on stirring the mixtures. All microemulsions were stored at ambient temperature. 

*Measurement of droplet size and zeta potential*

The average droplet size and zeta potential of the microemulsions were measured using a Zetasizer Nano-ZS (Malvern Instruments, UK). The measurement was performed at 25°C.

*In vitro permeation study*

The in vitro permeation rates of aceclofenac from various microemulsion formulations were determined to evaluate the effects of the formulation factors (19-23). 

The permeation experiments were performed using Keshary-Chien diffusion cells with 0.45-μm cellulose acetate membrane (Sartorius) at 37 ± 0.1°C using a thermostatic water pump (Cyberbath, CB 2000, Cyberlab Inc. USA). The effective diffusion area was 2.54 cm^2^ (18 mm diameter orifice), and the receptor compartment was filled with 13.5 mL of phosphate buffer at a pH of 7.4. The receptor fluid was constantly stirred by externally driven Teflon coated star head magnetic bars. 

Accurately weighed 1 g of aceclofenac was placed in the donor compartment. Samples (0.5 mL) were withdrawn from the receptor fluid at predetermined time intervals for upto 6 h after the beginning. An equal volume of the fresh phosphate buffer was immediately replenished after each sampling. All the collected samples were stored at -20°C until analysed by HPLC. The permeation study was performed in triplicate.

*Determination of the amount of aceclofenac by HPLC*

The amount of aceclofenac in receptor compartment was determined by HPLC. The HPLC system consisted of a pump (model Jasco PU-2080 plus, intelligent HPLC pump), a 20-μL loop sample injector (#7725i, Rheodyne, USA) , and a UV-Vis detector (Jasco UV-2075 intelligent UV-Vis detector model.) The equipment was operated by using the Borwin software, version 1.5, LC-Net II/ADC system. The column used was Inertsil ODS, C_18 _column having dimensions of 4.6 mmφ×250 mm i.d. and a particle size of 5 μm (GL. Sciences INC, JAPAN.)

The samples were chromatographed using an isocratic mobile phase consisting of a 50:50 v/v mixture of a 25 mM TRIS hydroxyl-methyl amino methane solution in phosphate buffer of pH 7.0, and acetonitrile. The pH of mobile phase was adjusted to 7.0. The flow rate was 1.5 mL/min and the detection wavelength was 276 nm. All operations were carried out at ambient temperature.

*Optical birefringence*

The formulations were examined by polarized light microscopy (Videoplan 11 UP, polarizing microscope, Japan) in order to determine the optical isotropy of the samples (14, 24). 

*Determination of pH*

The pH values of the samples were measured by a pH meter (model HI 8417, Hanna Instruments Inc., Woonsocket, USA), at 20±1°C.

*Viscosity measurement*

The viscosities of microemulsions were measured using a Brookfield rotational viscometer (LV2, Brookfield Inc., USA) equipped with the spindle no. 4. The measurement was performed at ambient temperature and in triplicate (14, 25-29).

*Statistical analysis*

All studies were performed in triplicate and the values were expressed as mean ± SD The data were analysed by one way analysis of variance (ANOVA) followed by Dunett test. A value of P < 0.05 was considered as significant. The “Graph Pad Instat Demo Version” software was used for the analysis of data.

## Results and Discussion

*Screening of oils, surfactants and co-surfactants for microemulsion formation*

To develop microemulsion formulations for topical delivery of the poorly water-soluble aceclofenac, the appropriate oil must be chosen. The solubilities of aceclofenac in various oils, 

surfactants and co-surfactants are shown in Table 2.

**Table 2:** Solubility of aceclofenac in different oils, surfactants and co-surfactants

**Phase type **	**Excipient**	**Solubility (mg/mL)**
Oil	Isopropyl myristate	2.94 ± 0.08
	Soy bean Oil	1.07 ± 0.13
Surfactant	Cotton seed Oil	1.45 ± 0.06
	Olive Oil 2	1.26 ± 0.12
	Labrasol	395.27 ± 2.77
	Tween80	399.21 ± 2.37
Co-surfactant	Cremophor RH – 40	272.91 ± 2.75
	Labrafac Lipofile	6.24 ± 0.34
	Poly Ethylene Glycol 400	48.60 ± 3.696
	Plurol Oleique	110.68 ± 3.48

The aceclofenac solubility in various oils investigated, was found to be the highest in isopropyl myristate (2.94±0.08 mg/mL), followed by cotton seed oil, olive oil and soya bean oil. Amongst surfactants, tween 80 showed the maximum solubility (399.21±2.37 mg/mL) followed by labrasol and cremophor RH-40. Plurol oleique showed the highest solubility among the co-surfactants (110.68±3.48 mg/mL), followed by polyethylene glycol 400 and labrafac lipophile.

Based on solubility studies of aceclofenac in the oils, surfactants and co-surfactants, and the preformulation studies ,it was found that IPM,and labrasol, plurol oleique could be the most appropriate combination for the microemulsion development of microemulsion.

*Construction of pseudo-ternary phase diagrams and microemulsion formulation*

**Figure 1:**
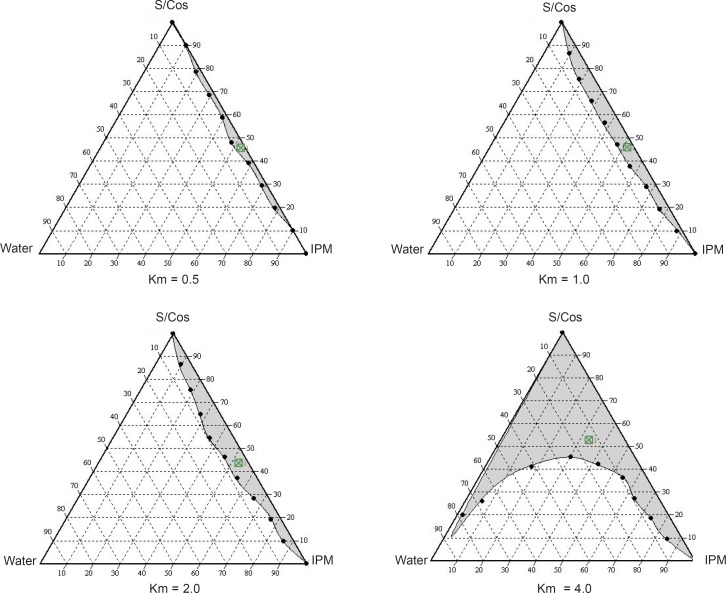
Pseudo-ternary phase diagrams for microemulsions composed of oil (Isopropyl Myristate), surfactant (S, Labrasol), co-surfactant (Co-S, Plurol Oleique) and water

Phase diagrams were constructed to determine the microemulsion regions. The pseudo-ternary phase diagrams with various weight ratios of labrasol to plurol oleique are presented in Figure 1. 

**Figure 2:**
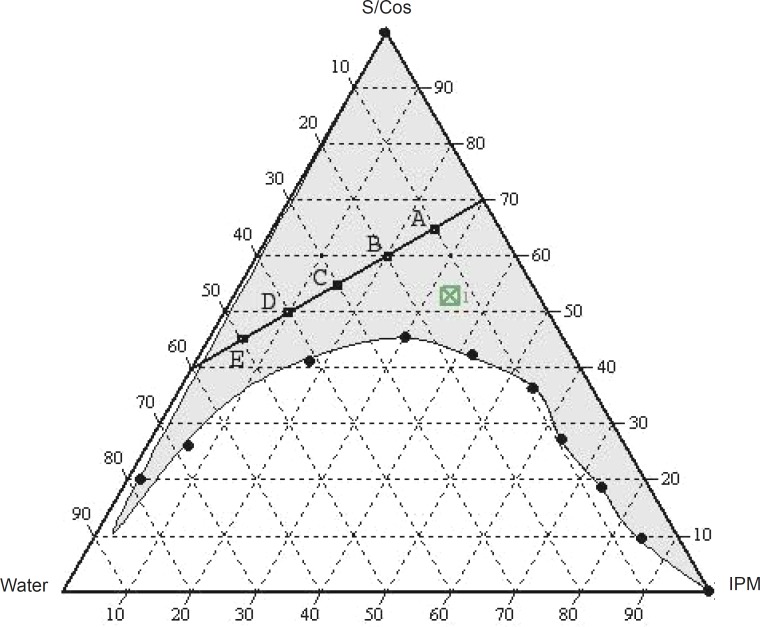
Pseudo-ternary phase diagram showing the different compositions of the microemulsion formulations

The translucent microemulsion region is shown in phase diagrams. The compositions of the different microemulsion formulations prepared are represented in Figure 2. 

No distinct conversion from water-in-oil (w/o) to oil-in-water (o/w) microemulsion was observed. The rest of the phase diagram represents the turbid conventional emulsion, based on visual inspection.

The phase study clearly reveals that with an increase in the weight ratio of surfactant (0.5-4), the microemulsion region is also expanded. The maximum proportion of oil was incorporated in a 4:1weight ratio of labrasol to plurol oleique. Varying proportions of surfactant - co-surfactant (65-45%), oil (25-5%) and water (10-50%) were selected for formulation. 

*Measurement of droplet size and zeta potential*

The droplet size and zeta potential of the formulations are represented in Table 3. The results show that the droplet size decreases withincreasing ratio of oil: surfactant/co-surfactant. These results are in accordance with the previous report that the addition of surfactant to microemulsion system caused the interfacial film to condense and become stable, while the co surfactant causes the film to expand (30). 

**Table 3:** Droplet size and zeta potential of formulations

Sr. No.	Formulation	Droplet size (nm)	Zeta potential (mv)
1	AILP – A	246	-0.232
2	AILP – B	213	-0.114
3	AILP – C	200	-0.535
4	AILP – D	144	-0.596
5	AILP – E	136	-0.185

The pH values and viscosities of formulations are listed in Table 4. 

**Table 4:** pH and viscosities of formulations

**Sr. No. **	**Formulation **	**pH **	**Viscosity **
1.	AILP – A	3.41	81.7
2.	AILP – B	3.25	90.0
3.	AILP – C	3.20	78.3
4.	AILP – D	2.90	81.7
5.	AILP – E	2.82	78.3

*In vitro permeation study*

**Figure 3:**
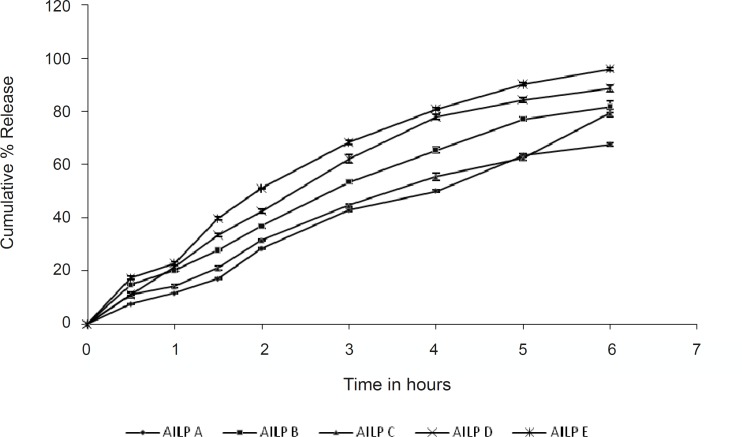
In vitro cumulative percent drug permeated from microemulsion formulations

The drug permeation rates from various microemulsion formulations are illustrated in Figure 3. Amongst the formulations tested, the batch AILP – E showed the highest permeation rate (95.88%). The content of the surfactants mixture in microemulsions significantly affected the permeation rate of aceclofenac. As the content of the surfactants mixture was decreased from 65% to 45% at S/CoS = 4, the permeation rate of aceclofenac increased by 2 folds. This may be due to an increase in thermodynamic activity of the drug in the microemulsion at the lower content of surfactant, as aceclofenac is poorly water soluble and is solublised in the surfactant mixture (31). The results of the statistical tests also revealed that the formulation AILP – E showed a significant difference as compared to the marketed formulation (P< 0.05), while the other formulations showed no significant difference. 

The particle size of the microemulsion droplets also affects the percutaneous absorption of the drug. When the droplet size is very small, there is a chance that the number of vesicles that can interact with a fixed area of stratum corneum to increase, thereby increasing the efficiency in percutaneous uptake. This might be the reason why the other microemulsions whose particle sizes were larger than that of the formulation AILP – E showed relatively lower permeation rates. 

From the permeation studies, it is clearly revealrevealed that the permeation rate increases (67% to 95 %), as the concentration of both the oil and the S/CoS mixture decreases. 

## Conclusion

For the formulation of microemulsions containing aceclofenac, the proper components and their optimum concentration ranges were obtained using pseudoternary phase diagrams. The concentrations of the main components were optimised after evaluation of their effects on drug permeation. The formulation AILP – E was considered as the optimised microemulsion consisting of IPM 5%, labrasol/plurol oleique 45% (4:1) and water.
